# The influence of glycation on a high pressure denaturation of ubiquitin

**DOI:** 10.1042/BSR20160233

**Published:** 2016-10-14

**Authors:** Monika Kijewska, Karolina Radziszewska, Marta Cal, Mateusz Waliczek, Piotr Stefanowicz, Zbigniew Szewczuk

**Affiliations:** * Faculty of Chemistry, University of Wrocław, Wrocław 50-383, Poland

**Keywords:** high-pressure denaturation, hydrogen–deuterium exchange, mass spectrometry, non-enzymatic glycation

## Abstract

The combination of deuterium–hydrogen exchange (DHX) and mass spectrometry (MS) can be used for studying a high pressure denaturation (HPD) of proteins. Herein we present the results of investigations of the influence of glycation on the HPD of ubiquitin. Application of various values of pressure causes different degrees of protein unfolding, resulting in molecules with a different number of protons available for exchange with deuterons. The dependence of this number on pressure gives information on the denaturation state of a protein. On the basis of the obtained results we can conclude that increasing number of fructosamine moieties in ubiquitin decreases the pressure required for its denaturation. It suggests that glycation moderately decreases the protein stability. The present study is the first example of application of hydrogen–deuterium exchange as a method of investigating the influence of posttranslational modification of protein on the HPD.

## INTRODUCTION

In the last decade, the food industry has shown a rising interest in the high-pressure (HP) or high hydrostatic pressure (HHP) technologies as non-thermal processes to achieve food safety and improve or maintain the quality of some products [1–3]. However, HHP can affect protein conformation, change its functionality and generate pressure transitions such as denaturation, aggregation or gelation, which depend on pressure level, temperature, type of protein and time [4]. On the other hand, it also becomes clear that along with such parameters as temperature and solvent conditions, pressure can be used for more detailed thermodynamic and kinetic description of bioprocesses and biosystems and regulation of their behaviour [5]. For example, in protein denaturation studies, HHP provides unique information on the unfolding mechanism [6]. High-pressure techniques have been applied in the studies on protein folding and, more recently, in the studies on the transition states of that process. Such studies require high-pressure techniques integrated with FTIR (Fourier-transform infrared), NMR, SAXS, densitometry, fluorescence and fourth derivative UV absorption spectra [7–11].

At high pressures (above 200 MPa), many proteins tend to unfold and reassociation of dissociated subunits from oligomers can occur. Under certain conditions, dissociation and/or denaturation induced by HP can be reversible [12,13]. Denaturation of proteins by high pressure differs from heat-induced denaturation, the latter being often irreversible because of the breakage of covalent bonds and/or aggregation of unfolded protein. Although the mechanism by which the protein is denatured by pressure is not yet fully understood, it has been suggested that HP causes changes in protein structure via cleavage of non-covalent bonds [12]. For example, partial unfolding of BSA has been observed at pressures as low as 200 MPa [14]. However, the protein is relatively stable up to 400 MPa due to its rigid and compact, globular structure stabilized by 17 disulfide bonds [15]. Nevertheless, similarly to other proteins, the changes in BSA structure largely depend on the magnitude and duration of the pressure applied. The effect of combined heat and pressure on the Maillard reaction between BSA and glucose was investigated. The kinetic results showed that the protein–sugar conjugation rate increased with increasing temperature, whereas it decreased with increasing pressure [16]. Recently very promising research has been performed to alter the structure of the allergen proteins by a high pressure to reduce its allergenicity. The structural changes in those proteins, studied by spectroscopic methods, were correlated with the observed allergenicity (IgE binding) changes [17].

In our recent work we investigated the influence of high pressure on the glycation level of a model protein–ubiquitin. The glycation is non-enzymatic reaction between the free amino group of a protein with the reducing sugar [18]. The biological aspect of glycation is widely discussed in literature [19–22]. For example, the non-enzymatic glycation of α-crystallin with reducing sugars has been reported to be one of the most significant factors leading to the age-related cataract, in particular for diabetic patients [23,24]. Moreover, the role of this reaction in preservation of food is very important because the simultaneous occurrence of sugars and proteins necessary for Maillard reaction in food systems is obvious. In our previous study we noticed that the level of glycation of the model protein under high pressure may be compared with the degree of modification under atmospheric pressure for the same time of incubation. In both cases, the slight increase in glycation was observed in contrast with thermal glycation [25].

In this work we wanted to expand our investigations and check the influence of glycation on the protein conformational stability (resistance to denaturation) under a high pressure using mass spectrometry (MS). We applied a method combining a deuterium–hydrogen exchange (DHX) and electrospray MS for studying a high-pressure denaturation (HPD) of proteins developed in our research team [26]. This method was successfully applied for amyloidogenic protein–the wild-type human cystatin C (hCC)–and its single-point mutants, in which the Val^57^ residue from the hinge region was substituted by asparagine, aspartic acid or proline respectively. Observation of H/D isotopic exchange occurring during pressure induced unfolding and subsequent refolding allowed to detect differences in the proteins stability and folding dynamics [27].

Our research was performed on the sample of glycated protein with the average glycation level 2.4, containing a free protein and its modified forms containing 1, 2, 3, 4 and 5 hexose moieties in a protein molecule. Our results demonstrated the increase in protein denaturation susceptibility with the glycation level which indicates that the structural stability of glycated protein is lower as compared with unmodified ubiquitin.

## MATERIALS AND METHODS

### Reagents

Deuterium oxide (99.9%D), acetic acid (HPLC grade), formic acid (HPLC grade), acetonitrile (LC–MS liquid chromatography MS grade), ammonium formate (LC–MS grade) and ubiquitin were purchased from Aldrich. D-Glucose was purchased from Eurochem. The reagents were used without further purification.

### High-pressure equipment

The HPD experiments and glycation under high-pressure were carried out using a custom-made cylinder-piston-type apparatus. The stoppered polypropylene syringe, containing the protein sample, was placed in a high-pressure vessel filled with hexane as a transmission medium and compressed (0.001–14 kbar).

### High temperature glycation

The high temperature glycation of ubiquitin was performed according to Boratyński method [28,29]. Briefly: protein and sugar were dissolved in water at the 1:10 protein to sugar molar ratio and lyophilized. The dry lyophilizate was placed in an air oven at 80°C for 25 min (according to the method described by us previously [30]). The resulting preparation was lyophilized and directly used for HPD experiments.

### High-pressure experiments

The solutions of 0.1 mg of a high temperature glycated ubiquitin in 0.1 ml of 10 mM ammonium formate in ^2^H_2_O (pD 6.6) were incubated at a given pressure (0.001–10 kbar) for 30 min at room temperature. After decompression samples were immediately diluted with 500 μl of 10 mM ammonium formate (pH 6.6) in water to initiate the DHX under atmospheric pressure. The compression and decompression times were approximately 2 min. The final concentration of a protein was 0.1 mg/ml. The obtained solution was directly infused into the ion source using a T connector. T connector was applied to introduce at the same time as the sample the mixture of acetonitrile and formic acid to enhance the ionization of the sample and to denature it.

### ESI-MS measurement

ESI-MS experiments were performed using an Apex-Qe Ultra 7T instrument (Bruker Daltonic, Germany) equipped with an ESI source. The instrument was operated in the positive-ion mode and calibrated with the TunemixTM mixture (Bruker Daltonic, Germany). The mass accuracy was better than 5 ppm. Spectra were recorded using aqueous solutions of acetonitrile (50%) and formic acid (1%), at a protein concentration 5 μM. The sample in the high-pressure experiment was infused at a flow rate of 3 μl/min. The obtained mass spectra were analysed using a Biotools (Bruker Daltonic, Germany) software. The instrumental parameters were as follows: scan range 300–2500 *m*/*z*; drying gas nitrogen; temperature of drying gas 200°C; the potential between the spray needle and the orifice 4.5 kV; source accumulation 0.5 s; ion accumulation time 0.5 s. Analysis and deconvolution of the obtained spectra were carried out with a Biotools (Bruker) software.

### Statistical data

Experiments using HPD of high temperature glycated ubiquitin combined with DHX and electrospray ionization MS were repeated twice. In all cases the obtained data showed the same values of denaturating pressure (±0.1 kbar). The statistical data was performed using the S.E.M.

## RESULTS AND DISCUSSION

High pressure denaturation combined with DHX and electrospray ionization MS was carried out on high temperature glycated ubiquitin. The main aim of these experiments was an investigation of the influence of this modification on the conformational stability of the protein and its denaturation.

**Figure 1:**
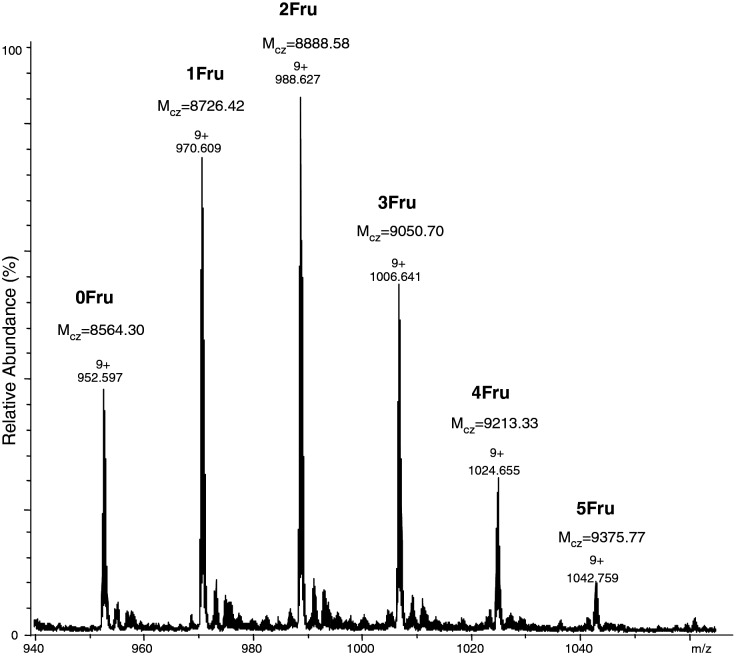
ESI-MS spectrum of glycated ubiquitin

The ubiquitin was thermally glycated according to Boratynski method [28–30]. The ESI-MS spectrum of glycated protein presented in Figure 1 shows that the number of hexose (fructosamine) moieties attached to the ubiquitin molecule ranges from 0 to 5. The average number of hexoses moieties per 1 molecule of ubiquitin (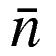), calculated according to formula (1) is 2.4.

1 $$\bar{n}=\frac{\sum_{n}nA_{n}}{\sum_{n}A_{n}}$$

where:

*n*–the number of hexose moieties attached to the ion;
*A_n_*–the abundance of the ion corresponding to ubiquitin with *n* moieties of hexose attached.

Glycation of ubiquitin results in a mixture of isomeric compounds that differ by the number and location of glycated lysine moieties which was confirmed by analysis of the ECD fragmentation of synthetic peptide-derived Amadori products, thermally glycated ubiquitin as well as its peptide fragments [31,32].

The sample of glycated ubiquitin was dissolved in 10 mM ammonium formate in deuterium oxide, pD 6.9, and subjected to a high-pressure treatment (HPT) at a given pressure (from the range 0.001–10 kbar) for 30 min at room temperature. After decompression, samples were immediately diluted with 500 μl of 10 mM ammonium formate (pH 6.6) in water to initiate the DHX under atmospheric pressure. The compression and decompression times were approximately 2 min. The obtained solution was directly infused into the ion source. Mass spectra were recorded every 2–3 min after introduction of the sample into the ion source, up to 80 min, and then the MS spectra analysis was carried out using DataAnalysis Compass (Bruker Daltonics) (deconvolution function). Very small temperature changes (Δ*T* less than 2°C) during decompression or compression were observed suggesting that the thermal effect do not influence the level of glycation or stability of ubiquitin.

In all experiments, the signal corresponding to a molecular mass of the modified protein was shifted towards lower masses during the measurement. Regardless of the value of the applied pressure, glycated ubiquitin molecule incubated in deuterated solvent was characterized by a substantial degree of H/D exchange. After 60 min of isotopic D/H back exchange, glycated ubiquitin still contained deuterons. The data obtained were analysed according to the procedure published previously by Stefanowicz et al. [26]. The average content of deuterium in a denatured protein was calculated as the difference in the average mass between the denatured and the native protein and the number of attached hexose moieties (Fru) by eqn (2):

2 $$D\left(r\right)=M_{D}-M_{0}-n*Fru$$

where:

*D*(*r*)–the number of remaining deuterons;
*M*_D_–the observed average molecular mass of a denatured protein;
*M*_0_–the average molecular mass of a non-denatured protein (non-modified ubiquitin);
*Fru*–the molecular mass of aminofructose (162.053 Da);
*n*–the number of Fru attached to ubiquitin (*n*=1, 2, 3, 4, 5).

**Figure 2:**
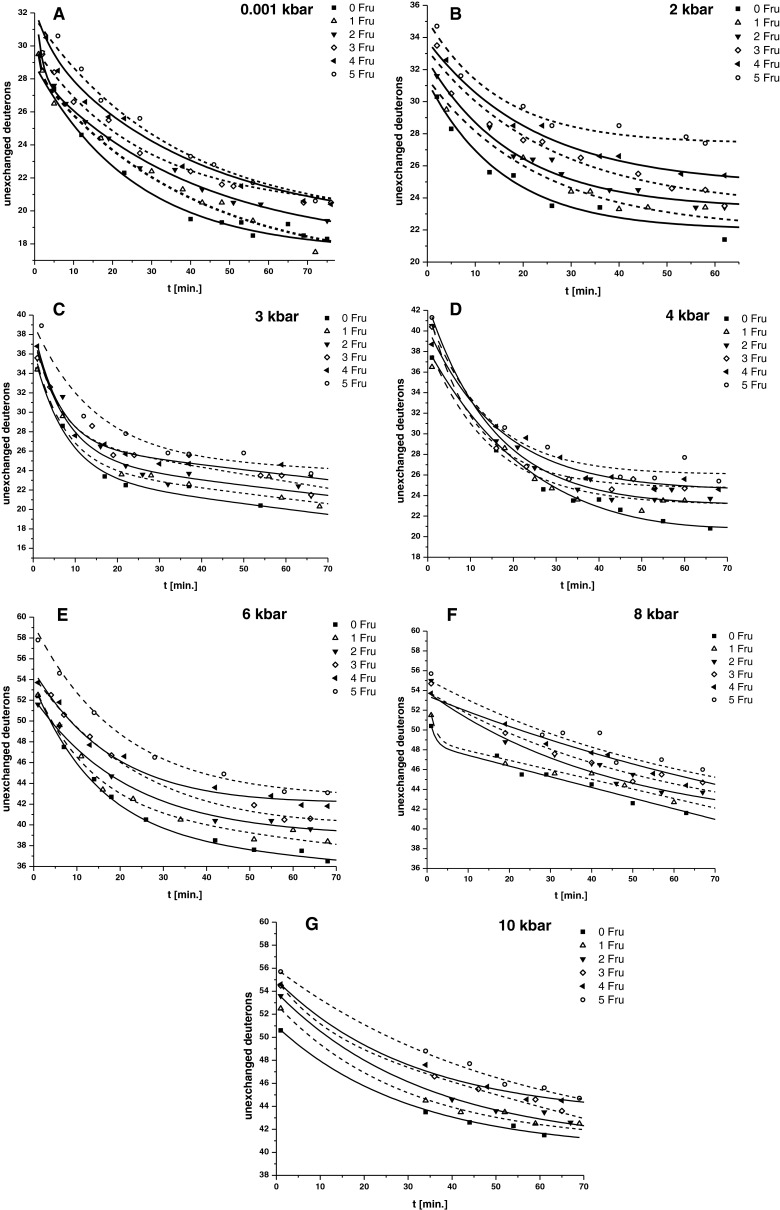
DHX kinetics of modified ubiquitin treated with ^2^H_2_O as a function of time for various pressures (The number of unexchanged deuterons in the molecule of modified ubiquitin as a function of the back exchanged D/H for samples incubated under different pressures).

In Figures 2(A)–2(G) the DHX kinetics of modified ubiquitin treated with ^2^H_2_O at various pressures is presented. The curves show the dependence of unexchanged deuterons in modified ubiquitin on the D/H back exchange time. It is the exponential dependence described by equation (3):

3 $$D_{\left(r\right)}=D_{1}\exp\left(-k_{1}t\right)+D_{2}\exp\left(-k_{2}t\right)+D_{3}$$

where:

*D*(*r*)–the number of unexchanged deuterons;
*D*_1_–the number of deuterons undergoing a very rapid isotopic exchange
  (the exchange rate constant *k*_1_);
*D*_2_–the number of deuterons undergoing a slow isotopic exchange (the exchange rate constant *k*_2_);
*D*_3_–the number of deuterons unexchangeable, completely protected from isotopic and back exchange.

In Figure 2 we can observe that regardless of the HPT the curves have a similar shape and after approximately 60 min of back exchange in the aqueous buffer there remains a constant number of unexchangeable deuterons in the molecule of modified ubiquitin. At atmospheric pressure glycated ubiquitin occurs in the native form so the only possible H/D exchange can take place on the surface of the protein molecule.

After 60 min of back exchange in the aqueous buffer under pressure 6 kbar, approximately 20 deuterons remained in the protein molecule depending on the number of attached fructose moieties (for ubiquitin it is 18 and for ubiquitin substituted with 5 Fru's it is 22). Undoubtedly, during a 5-fold dilution of the buffer solution containing D_2_O with the buffer containing H_2_O, there is 20% 2H-labelled water which may explain the presence of some of the deuterons in ubiquitin. The remaining deuterons are probably located in the hydrophobic core of the protein. Their number should not depend on the degree of protein glycation because the Fru moiety should appear on the surface of the protein molecule. The exchange of deuterons for these protons often requires longer incubation times. Data obtained for ubiquitin substituted with 1–5 Fru residues allowed for identification of the additional amount of non-exchanged deuterons in modified protein (approximately 4 for ubiquitin containing 5 Fru's). This value corresponds to the expected number of deuterons which remain on fructosamine residues of ubiquitin placed in a solution containing 20% D2-labelled water. This result suggests that glycation did not significantly affect the conformational stability of ubiquitin at atmospheric pressure.

**Figure 3:**
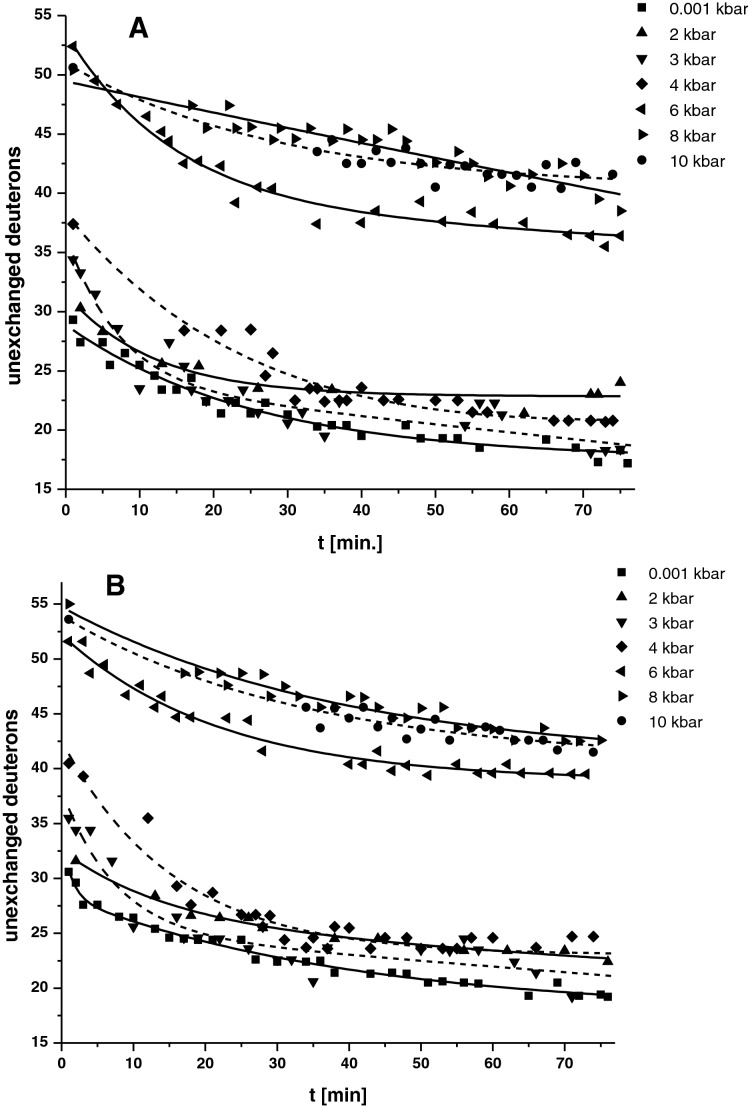
DHX kinetics of (A) unmodified and (B) diglycated ubiquitin treated with ^2^H_2_O at various pressures (The number of unexchanged deuterons in the molecule of modified ubiquitin as a function of the back exchanged D/H for samples incubated under 0.001–10 kbar).

Increased pressure causes an increase in the degree of exchange of protons for deuterons in a glycated protein molecule (Figure 3). After 60 min of the D/H back exchange, modified ubiquitin molecules still show a significant amount of trapped deuterons. The sample of the protein incubated under a high pressure presents a higher content of deuterium (more than 55 deuterons) for the sample incubated under a pressure of 10 kbar than the protein incubated in the denaturating buffer at the atmospheric pressure (30 deuterons only) (Figures 2A and 2G). After decompression and refolding process, deuterons are trapped inside the molecule, preventing their further back exchange. Further penetration of solvent molecules into the protein structure is difficult–the number of deuterons remaining inside was determined to be constant at approximately 40. Pressure below 4 kbar did not influence the content of deuterium in the protein sample. Renatured ubiquitin, previously denatured at the pressure of 6 kbar, entraps approximately 20 deuterons more than the protein denatured at 4 kbar. A further increase in pressure had very little effect on the number of additional deuterons trapped in the hydrophobic core of the protein. This proves that ubiquitin and its modified analogues denature under pressure above 6 kbar, resulting in the exchange of protons previously not exposed on the surface of the molecule. Similar experiments carried out at the 0.001–4 kbar pressures show that both ubiquitin and its glycated analogues show a similar stability at 4 kbar. Further increase in pressure causes a significant increase in the degree of H/D exchange in the molecule, both for unmodified and glycated ubiquitin, which means that at higher pressures we can observe denaturation of ubiquitin and its derivatives, followed by their renaturation after decompression.

**Figure 4:**
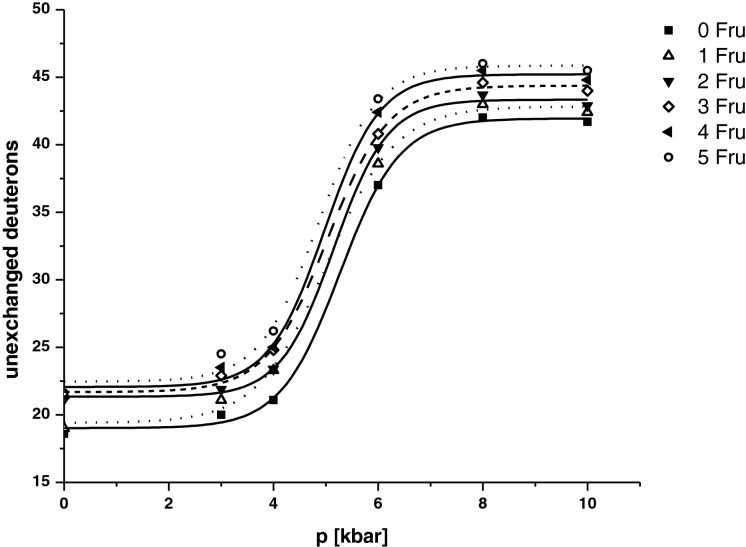
High-pressure denaturation of modified ubiquitin Graph of the number of deuterons trapped in the hydrophobic core of the protein after HPT followed by 1 h back exchange in the aqueous buffer as a function of the applied pressure. Denaturating pressures calculated from the Boltzmann sigmoidal equation: 0Fru–5.27 kbar; 1Fru–5.01 kbar; 2Fru–5.15 kbar; 3Fru–5.03 kbar; 4Fru–4.97 kbar; 5Fru–4.85 kbar.

Changes in the content of deuterium in protein molecules were plotted as a function of pressure. These graphs provide information on the denaturation of the modified ubiquitin under a high pressure. The number of deuterons trapped in the hydrophobic core of the substituted ubiquitin after HPT, followed by the 1 h back exchange in the aqueous buffer, as a function of the applied pressure (denaturation curves) are presented in Figure 4. The curves were fitted to the sigmoidal Boltzmann function:

4 $$y=\frac{A_{1}+A_{2}}{1+\exp[\left(x-x_{0}\right)/B]}+A_{2}$$

A similar equation was used by Powel and Fitzgerald [33] to describe the HDX under denaturing conditions.

**Figure 5:**
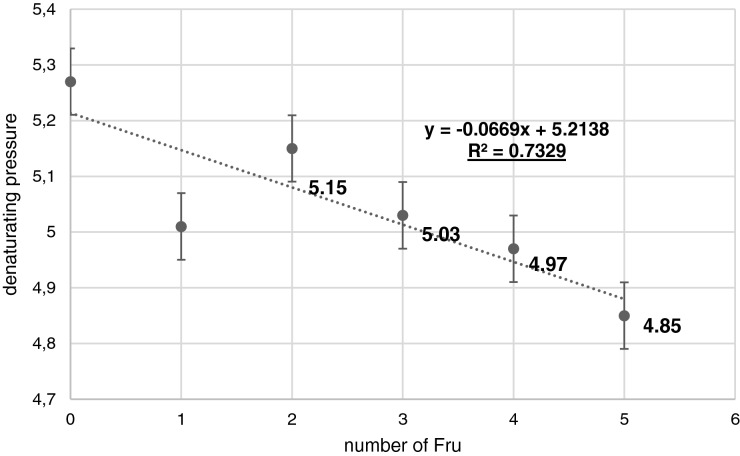
Dependence of the denaturating pressure for substituted ubiquitin in the H/D exchange experiment on the number of hexose moieties attached to ubiquitin

Only one inflection point on the sigmoidal plot suggests that unfolding of ubiquitin occurs in one step, with the midpoint at approximately 5.3 kbar. This result is in good agreement with the results obtained by Jonas et al. [34] (5.4 kbar) and Stefanowicz et al. [26] (5 kbar) as well as the data obtained by Herberhold and Winter [35] for ubiquitin using high-pressure FTIR. Analysis of individual curves corresponding to protein substituted by 1, 2, 3, 4 and 5 Fru's showed that increasing number of hexose moieties in ubiquitin decreases its pressure of denaturation, suggesting that this modification decreases the stability of the protein. Ubiquitin shows a very simple denaturation curve with the midpoint at approximately 5.3 kbar and the corresponding values for its glycated analogues showed in Figure 4. The obtained data are presented in Figure 5. The conducted experiments showed that the pressure required for protein unfolding (Y axis) correlates linearly with the number of hexose moieties attached to the ubiquitin (*X*-axis). The plot shown in Figure 5 has a high correlation coefficient equals to 0.73. The obtained results unambiguously suggest that with an increasing number of hexose moiety in ubiquitin decreases the pressure of denaturation. Similar approach was applied previously in denaturation studies of an amyloidogenic protein–the wild-type hCC and its single-point mutants, in which the Val^57^ residue from the hinge region was substituted by asparagine, aspartic acid or proline. Polar asparagine does not influence the stability of hCC conformation significantly, whereas charged aspartic acid at position 57 makes the protein structure slightly more prone to unfolding [27].

## CONCLUSION

We applied HPD of high temperature glycated ubiquitin combined with DHX and electrospray ionization MS for investigation of the influence of posttranslational modification on the conformational stability and denaturation. Our results show that the increasing number of fructosamine moieties attached to ubiquitin decreases the pressure required for its denaturation, suggesting that this modification moderately decreases the stability of the protein. Moreover, analysis of the mixture of non-modified ubiquitin and ubiquitin derivatives modified by 1, 2, 3, 4 or 5 fructosamines in one experiment eliminates the errors that may result from independent samples, dilution and measurement conditions. It is the first application of MS as a method of studies on the HPD of posttranslationally modified proteins. This method may be applied for the analysis of influence of different posttranslational modifications on denaturation stability of proteins treated with chemical agents. The proposed approach is a good alternative to currently used methods for testing the modified protein unfolding.

## Abbreviations

DHX: deuterium–hydrogen exchange
HHP: high hydrostatic pressure
HPD: high pressure denaturation
HPT: high-pressure treatment
MS: mass spectrometry
